# Improving early administration of antibiotics: a 'Plan Do Study Act' approach

**DOI:** 10.1186/cc10662

**Published:** 2012-03-20

**Authors:** A Revill, N Wennicke, J Tipping, R Matull

**Affiliations:** 1 Derriford Hospital, Plymouth, UK; 2 Taunton Musgrove Park Hospital, Taunton, UK

## Introduction

Delayed administration of antibiotics is associated with an increased mortality in severe sepsis. The Surviving Sepsis Campaign advocates administering antibiotics to severely septic patients within 1 hour. Predicting the patients that will become severely septic is difficult, and therefore we have introduced a pathway via a unique care bundle to identify and treat all patients with suspected sepsis, prior to significant organ dysfunction, and maintain a 1-hour target.

## Methods

In September 2009, we introduced an audit proforma and management tool into the medical admissions unit of our hospital. This was accompanied by an extensive education programme of all medical and nursing staff. The proforma consists of two parts, a recognition and intervention section. The process is triggered when the patient satisfies two of the SIRS criteria and has symptoms consistent with an infection. All six management processes, including antibiotic administration, must then be completed within 1 hour of the trigger time. By using the 'Plan Do Study Act' cycle, we refined the proforma and streamlined the process and introduced it into emergency department and the surgical admissions unit. A dedicated multidisciplinary team was assigned to review and improve performance every 2 weeks by amending the form and processes.

## Results

**Figure 1:**
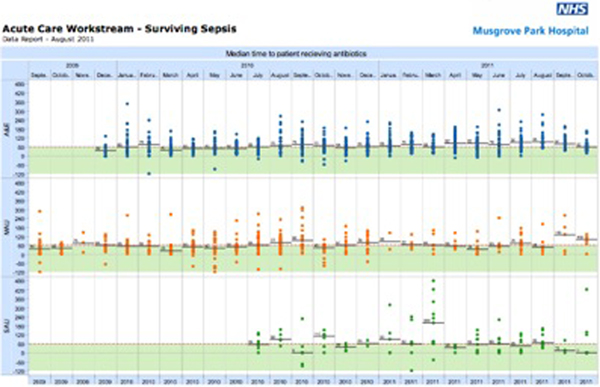
**Median time to antibiotics**.

Over a 24-month period we have a database with 1,571 patients. The results demonstrate that the median time to antibiotic administration is consistently near our target of 1 hour for all septic patients included in this pathway. Through continued refinement and staged introduction the proforma and the process has demonstrated consistency from medical to surgical wards; introduction in new areas has rapidly improved results. See Figure 1 overleaf.

## Conclusion

Our pathway has undergone a successful and dynamic development process guided by a multidisciplinary team. Compared with the usual audit process this has allowed rapid changes and improvements to take place and be tested. Further analysis of our database is ongoing, determining our impact on length of stay, mortality and intensive care admissions with a matched cohort.

